# The Use of Medical Imaging Request Forms as Trigger Tools to Detect Intra-Hospital Adverse Events: A Pilot Study

**DOI:** 10.5334/jbsr.2897

**Published:** 2022-11-10

**Authors:** Melody Saikali, Marin Halut, Antoine Saab, Bruno Vande Berg, Nicolas Michoux, Charbel Mourad

**Affiliations:** 1 Hopital Libanais Geitaoui, CHU, LB; 2 Cliniques Universitaires Saint Luc, BE; 3 CHC Montlégia, BE

**Keywords:** adverse events, trigger tools, Doppler ultrasound, chest radiographs

## Abstract

**Aim:**

To evaluate the contribution of medical imaging request forms as trigger tools to detect patient adverse event (AE) occurring during hospitalization.

**Material and Methods:**

This is a retrospective study in a single institution. Between January and June 2019, the hospital information system (HIS) was fetched for request forms of radiological examinations performed for inpatients >48 hours after the admission date. The investigated request forms were: Doppler ultrasound of the upper limbs, Doppler ultrasound of the lower limbs, and the repetition of three consecutive requests of chest radiographs within 24 hrs, to detect upper or lower limb venous thrombosis, or AEs related to the respiratory system, respectively. Patients’ medical charts and radiological examinations were evaluated to document the presence or absence of an AE. The frequencies of AEs in the three groups of trigger tools were compared to corresponding control groups, matched according to age, sex and length of stay.

**Results:**

Among a total of 2798 hospital admissions during the study period, there were 74 files triggered by the three types of radiological request forms. There were 6/24 AE (25%) related to upper limb venous thrombosis, 4/33 (12.1%) AE related to lower limb venous thrombosis, and 6/17 (35.3%) AE related to the respiratory system. For all the trigger tools, the frequency of AE in the study groups was significantly higher than that in the control groups.

**Conclusion:**

Medical imaging requests could be used as potential trigger tools to detect adverse events related to hospital stay.

## Introduction

An adverse event (AE) is defined as any injury caused by medical management rather than by the underlying disease or condition of the patient and does imply harm, which could necessitate further treatment or be fatal [1]. Although the concept of AEs is well established and has been documented for over two decades, they still remain to be the third cause of mortality in the USA [2,3]. This can partially be attributed to the lack of accurate information about the true incidence and nature of these AEs. In fact, the most commonly used method to detect AEs in hospitals has been through voluntary incident reporting systems, where approximately 1–5% of AEs are truly reported. In addition, self-reporting tools are used by patients in the USA and Europe to declare AEs [4,5].

The standard of reference for detecting AEs is the review of patient medical records. However, this is a resource intensive, retrospective method which does not allow for any real-time interventions. An alternative approach to is through the use of trigger tools (TT), which consists of a targeted analysis of flagged patient files based on certain triggers associated with the patient’s case, which can indicate a potential AE [6].

With the increased use of electronic medical records, detection of AEs via the TT method became easier by applying different filters on a large amount of data present in the Hospital Information System (HIS), to flag the medical files that could potentially harbor an AE. This reduces the number of medical files to review and increases the detection of AEs in a less resource intensive manner. Pilot studies using the TT method have been published, mainly related to intensive care units and pharmaco-vigilance [7]. The Institute of Healthcare Improvement (IHI) has developed a methodology using triggers that may detect a broad spectrum of AEs, namely the Global Trigger Tool (GTT) [1,8]. It relies on electronic triggers that already exist in the HIS, such as laboratory values, ordering of certain medications, readmissions, and the like. However, few have addressed the use of medical imaging requests as a TT to help detect AEs. We hypothesize that Doppler ultrasounds of the upper and lower extremities, and the repetition of chest radiographs ≥3 times/24 hours could be used as TT to detect intra-hospital deep venous thrombosis or respiratory complications, respectively.

## Materials and Methods

**Population:** This is a retrospective observational study conducted in a university tertiary hospital in Beirut, Beirut-Lebanon, aiming to identify patients with hospital acquired AEs using medical imaging trigger tools. This study was approved by the hospital’s Institute Review Board (IRB) and patient’s consent being waived.

**Figure 1:**
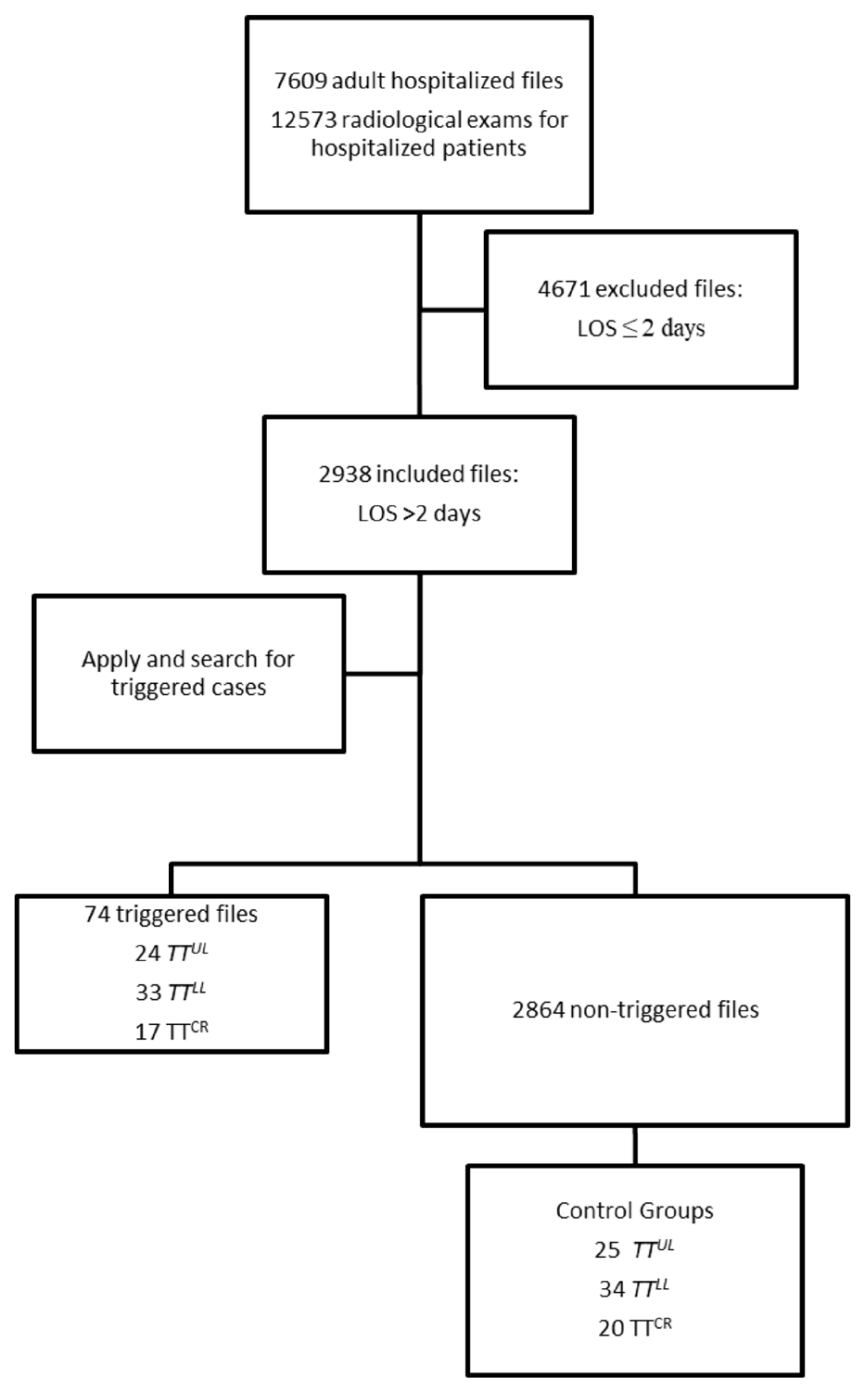
Flowchart summarizing the selection of triggered charts and control group. LOS: Length of Stay; TT^UL^: Trigger tool Upper Limb Venous Doppler Ultrasound; TT^LL^: Trigger Tool Lower Limb Venous Doppler Ultrasound; TT^CR^: Trigger Tool Chest Radiograph.

During the period of January–June 2019, a total of 7,609 adult patients were admitted (3,808 males) among which 2,938 (1,496 males) who had a length of stay ≥2 days were included in the study. Pediatric patients (<18 yrs), patients hospitalized for one day and psychiatric patients were excluded. The median age was 69 years (68–70 years, confidence interval (CI), 95%) and the median length of stay was 6 days (6–6 days, CI 95%) (Table 1). The inclusion process is summarized in the flowchart (Figure 1). During the same period, a total of 12,576 medical imaging examinations were performed for hospitalized patients. Radiological examinations were requested based on the clinical assessment of the prescribing physician.

**Table 1:** Demographic characteristics of the included patients.


		ADULT INPATIENTS			

		TOTAL	MALE	FEMALE	*p*-VALUE

	**Number (%)**	2938 (100%)	1496 (50.9%)	1442 (49.1%)	0.3282

**Age**	**Median (CI 95%) [min–max]**	69 (68–70) [18–102]	68 (67–69) [18–100]	70 (69–71) [18–102]	<0.05

**Length of Stay**	**Median (CI 95%) [min–max]**	6 (6–6) [3–212]	6 (6–6) [3–197]	6 (5–6) [3–212]	>0.05

**Department**		**Medicine/Surgery/** **Intensive Care/Other**		1308/1364/165/101 (44.5%/46.4%/5.6%/3.5%)	


### Definition of the Trigger Tools (TT) and Adverse Events (AE)

Three types of imaging request forms were selected as trigger tools (TT) by a multidisciplinary team composed of a senior radiologist, a radiology resident and two healthcare professionals from the quality and patient safety department. We aimed to detect the specific adverse events (AE) that have occurred >2 days after the hospitalization.

The imaging exam requests are:

- Doppler Ultrasound of the Upper Limb (TT^UL^) looking for venous thrombosis of the upper limbs (AE^ULVT^)
- Doppler Ultrasound of the Lower Limb (TT^LL^) looking for venous thrombosis of the lower limbs (AE^LLVT^)
- Repeated chest radiographs (at least three) within 24 hrs (TT^CR^), to potentially detect in hospital respiratory adverse events, for example a central line insertion associated pneumothorax or hospital-acquired pneumonia (AE^RC^)

### Study and Control Groups

Database of the hospital information system was fetched to select cases of inpatients, hospitalized for >2 days (n = 2938) and for whom a Doppler ultrasound of the upper extremities, lower extremities or repeated chest radiographs were requested. For each of the three groups of triggered cases, a control group counterpart was created including age and sex-matched individuals with similar lengths of stay.

### File analysis and classification of AE

The medical files were analyzed by a senior patient safety officer with seven years of experience in analyzing charts to detect AEs, and who was blinded to the patient’s group. The analyzed documents consisted of numerical data (e.g., laboratory values) and scanned paper-based documents. The imaging files were analyzed by a radiologist with four years of experience. The standard of reference for the presence or absence of an AE was a consensus between both investigators.

The harm severity level of the detected AEs were classified and stratified according to the NCC MERP index (National Coordinating Council for Medication Error Reporting and Prevention) [9].

The incident reporting system was reviewed for the duration of the study to detect if any adverse events related to the studied trigger tools were spontaneously reported by healthcare workers or patients.

### Statistical Analysis

The normality of distribution was assessed using Shapiro-Wilk test. The Mann-Whitney U Test and Figher’s exact test were used to compare the medians and proportions, respectively. A *p* value threshold of <0.05 was used for statistical significance. Statsdirect software (v3.1.20) was used for statistical analysis.

## Results

### Incidence of AEs detected using the TT

Among the 2,938 admissions, 74 files (2.5%) were triggered for review using the three TTs. Among the 74 triggered files, 16 AEs, from 16 files, were detected (21.6 %), resulting in an incidence of 0.54% (16/2938). No AE corresponding to the three triggers were reported using the incident reporting system.

### Detection of AE ^ULVT^ using TT^UL^

Six AE^ULVT^ were detected in a total of 24 triggered files (25%) (Table 2). No venous thrombosis was detected in the control group. This difference was statistically significant (*p* < 0.01). All of the detected AE^ULVT^ are classified as Category E according to the NCC MERP scale (temporary harm requiring intervention) (Supplementary File 1).

**Table 2:** Rate of detection of adverse events and demographic characteristics of patients in the study and control groups.


TRIGGER TOOL	DEMOGRAPHIC CHRACTERISTICS	STUDY GROUP (TRIGGERED FILES)				CONTROL GROUP			*p*-VALUE **
	
		TOTAL	AE+	AE–	P*	TOTAL	AE+	AE–	

**Doppler ultrasound of the upper limb**	**No. Of Cases** **(No. Of patients)**	24 (23)	6 AE^ULVT^ (5)	18 (18)	25 (25)	0	25 (25)	P = 0.0096

	**Median Age** **(CI 95%)** **[min–max]**	75.5 (68–79) [36–96]	77 (55.4–85) [55–86]	70 (65–79) [36–96]	P = 0.7872	72 (67–77) [54–90]	NA	72 (67–77) [54–90]	P = 0.984

	**Sex (M/F)**	15/9	4/2	11/7	P = 1	14/11	NA	14/11	P = 1

	**Department (Medicine/Surgery/ICU/Other)**	16/6/2/0	6/0/0/0	10/6/2/0	12/9/4/0	NA	12/9/4/0	

	**Median LOS** **(CI 95%)** **[min–max]**	15.5 (10–35) [3–187]	52.5 (7.7–187) [5–187]	12 (9–27) [3–185]	P = 0.1031	27 (11–30) [5–212]	NA	27 (11–30) [5–212]	P = 0.8415

	**Days before AE:** **Median (CI 95%) [min–max]**	NA	23.5 (6.7–100.7) [4–117]	NA	NA	NA	NA	

**Doppler ultrasound of the lower limb**	**No. Of Cases** **(No. Of patients)**	33 (33)	4 AE^LLVT^ (4)	29 (29)	34 (34)	0	34 (34)	P = 0.0267

	**Median Age** **(CI 95%)** **[min–max]**	70 (63.8–78.2) [20–90]	53.5 (NC) [30–90]	72 (64.5–79.5) [20–88]	NC	73 (67–77) [25–90]	NA	73 (67–77) [25–90]	P = 0.395

	**Sex (M/F)**	16/17	1/3	15/14	0.6012	18/16	NA	18/16	P = 0.8086

	**Median LOS** **(CI 95%)** **[min–max]**	10 (7–20) [3–197]	25.5 (NC) [8/89]	9 (6–20) [3–197]	NC	20 (12–29) [5–212]	NA	20 (12–29) [5–212]	P = 0.0784

	**Department (Medicine/Surgery/ICU/Other)**	16/13/3/1	2/1/1/0	14/12/2/1	19/10/4/1	NA	19/10/4/1	

	**Days before AE:** **Median (CI 95%) [min–max]**	NA	5.5 (NA) [5–15]	NA	NA	NA	NA	

**Repeated chest radiographs**	**No. Of Cases** **(No. Of patients**	17 (14)	6 AE^RC^ (6)	11 (11)	20 (20)	0	20	P = 0.0053

	**Median Age** **(CI 95%)** **[min–max]**	73.5 (57–80) [30–87]	72 (40–87) [40–87]	77 (57–84) [30–87]	1	76.5 (67.3–83.7) [25–87]	NA	76.5 (67.3–83.7) [25–87]	P = 0.5287

	**Sex (M/F)**	8/6	2/4	8/3	0.1618	13/7	NA	13/7	P = 0.7282

	**Median LOS (CI 95%)** **[min–max]**	18.5 (14–25) [5–70]	23 (18–70) [1–70]	16 (12–27) [5–70]	0.1585	20 (14.5–29.7) [6–87]	NA	20 (14.5–29.7) [6–87]	P = 0.4965

	**Department (Medicine/Surgery/ICU/Other)**	1/4/9/0	1/1/4/0	0/4/7/0	9/7/3/1	NA	9/7/3/1	

	**Days before AE:** **Median (CI 95%)** **[min–max]**	NA	13.5 (6–17) [6–17]	NA	NA	NA	NA	


AE+: Occurrence of an Adverse Event (AE). AE–: Absence of an AE. AE^ULVT^: Adverse Event of an Upper limb venous thrombosis. AE^LLVT^: Adverse Event of a Lower limb venous thrombosis. AE^RC^: Adverse Event of a Respiratory complication. CI: Confidence Interval; ICU: Intensive Care Unit; LOS: Length of Stay.

### Detection of AE^LLVT^ using TT^LL^

Four AE^LLVT^ were detected in a total of 33 triggered cases (12.1%) (Table 2). No venous thrombosis was detected in the control group. This difference was statistically significant (*p* < 0.01). One AE^LLVT^ level of harm was classified as Category E, and three were classified as Category F (temporary harm to the patient and required initial or prolonged hospitalization) (Supplementary File 1). The overall incidence of AEs related to venous thrombosis (upper and lower limb) using the Doppler ultrasound requests triggers was 10/2938 = 0.34%.

### Detection of AE^RC^ using TT^CR^

Six AE^RC^ were detected in a total of 17 triggered cases (35.3%). No AE^RC^ was detected in the corresponding control group (*p* < 0.01) (Table 2). All the detected AEs were classified as Category F (Supplementary file 1).

## Discussion

In the current study, we demonstrated that medical imaging request forms may be used as trigger tools (TT) to detect intra-hospital adverse events (AEs) with significant levels of harm (Categories E and F), and a positive detection rate ranging from 12–35%.

The current study is the first to evaluate the incidence of hospital-acquired venous thrombosis of the upper and lower limbs, in the Middle East region. The incidence of AE^ULVT^ and AE^LLVT^ detected in our population (0.34%) is comparable to the study of Khan et al. [10] (0.4%) that was performed on a similar population (Supplementary File 2). However, it is inferior to the incidence of venous thrombo-embolisms in the studies of Jenkins et al. [11] (0.9%) and Assareh et al. [12] (1.1%), which could be explained by the fact that they included pulmonary embolisms in addition to venous thrombosis. The incidence of venous thrombosis in the current study is superior to that of Khanna et al. [13] (0.25%), probably explained by the fact that they excluded surgical patients.

Our results demonstrate that the repeated prescription of chest radiographs could be used as a trigger tool to detect in-hospital respiratory AEs. This trigger tool could be incorporated into an automated system that integrates radiological data in addition to data extracted from the HIS (laboratory data, ICD codes, etc.) for a broader spectrum of detected AEs.

In the future, the ‘trigger tool’ method could be enhanced with artificial intelligence (AI) and clinical decision support tools that optimize inclusion and exclusion criteria, add to the quality of medical information, target more specific types of AE, and could improve the positive predictive value and incidence detection rates. As an example, the performance of the TT could be further improved by the use of natural language processing to reduce the number of cases to be analyzed.

Our study has several limitations. First, it was conducted in a single institution. Therefore, the performance of the TTs is dependent on the specific practice of the institution (e.g., performing Doppler studies by the radiologists and not by sonographers or vascular surgery department), and the effectiveness of the triggers is influenced by the prescribing habits of the physicians. A fortiori, the number of AEs in the control groups could therefore be underestimated since the absence of Doppler examinations could be linked to poor practice rather than to a low occurrence of thrombosis. The true incidence of venous thrombosis would have been only obtained by performing a systematic Doppler ultrasound for all the patients, which is beyond the scope of the current study. Second, the number of venous thrombosis AEs also depends on the diagnostic performance of the Doppler ultrasound (Sn and Sp 95%) and the operator [14]. However, the Doppler ultrasound is still considered to be the first line diagnostic tool to evaluating venous thrombosis events. In fact, the use of alternative diagnostic tests (such as the venography or the venous CT angiography) as potential trigger tools would not be as effective since they are usually performed as second line tools. On the other hand, the AE^RC^ are not affected by the diagnostic performance of the chest radiographs. Third, we only included patients who were hospitalized for more than two days in order to exclude one-day admission patients (chemotherapy, one day surgery, etc.). This inclusion criterion has been previously reported in the literature [3,11,13]. However, it can be modified according to the practices of each institution. Specific to TT^CR^, the inclusion criteria such as the number of consecutive radiographs or the time interval between the radiographs could be tailored in every institution in order to improve the positive predictive value. Fourth, the investigators who analyzed the records were not completely blinded to the patient’s group because the HIS and the PACS might contain information that could reveal the patient group. Finally, our results may underestimate the number of thromboses associated with hospitalization, because these, by definition, can develop 90 days after the end of hospitalization [10] and the post-hospitalization period was not included in the current study.

Finally, our results open the perspectives to compare the performance of TT based on radiology request forms with other TT, for example based on laboratory data and microbiology results. In addition, they could be implemented in software that enables automated detection of AEs in a near real-time manner.

In conclusion, Doppler ultrasound examinations of the upper or lower extremities and repeated chest radiographs can be used as trigger tools to detect adverse events related to hospitalization. Further studies are needed to confirm our preliminary data in other institutions.

## Additional File

The additional file for this article can be found as follows:

10.5334/jbsr.2897.s1 Supplementary File. Supplementary Files 1 and 2.
