# Supplementary material for: The Use of Medical Imaging Request Forms as Trigger Tools to Detect Intra-Hospital Adverse Events: A Pilot Study
Source: J Belg Soc Radiol. 2022 Nov 10;106(1):106. doi: 10.5334/jbsr.2897 (PMC9650978; doi:10.5334/jbsr.2897)
Supplement: Supplementary File. — Supplementary Files 1 and 2. [file jbsr-106-1-2897-s1.pdf]

Supplementary file 1: Classification of the 16 detected Adverse Events according to category and level of harm

Trigger Tools	AE Detected	Florida Hospital AE/Harm Category*	Level of Harm**
TT^{UL}	6 ULVT necessitating treatment without prolonged hospitalization	**Events Related to Patient Care**: VTE	**Category E**- An error occurred that may have contributed to or resulted in temporary harm to the patient and required intervention
TT^{LL}	1 LLVT necessitating treatment without prolonged hospitalization	**Events Related to Patient Care**: VTE	**Category E**- An error occurred that may have contributed to or resulted in temporary harm to the patient and required intervention
	3 LLVT necessitating treatment without prolonged hospitalization	**Events Related to Patient Care**: VTE	**Category F**- An error occurred that may have contributed to or resulted in temporary harm to the patient and required initial or prolonged hospitalization
TT^{CR}	1 pneumothorax due to tube malpositioning	**Events Related to Surgery or Other Procedure:** Respiratory complications related to surgery or procedure.	**Category F**- An error occurred that may have contributed to or resulted in temporary harm to the patient and required initial or prolonged hospitalization
	1 respiratory acidosis post cholecystectomy		
	1 pre-mature extubation causing re-intubation and escalation in level of care	**Events Related to Surgery or Other Procedure:** Premature extubation causing respiratory failure	
	1 pneumonia post Whipple surgery	**Hospital Acquired Infections:** Postoperative respiratory infection	
	1 pneumonia post cardiac surgery		
	1 hospital acquired pneumonia	**Hospital Acquired Infections:** Respiratory infection (non-ventilator associated)	

* Florida Hospital AE/HarmClassifcation(1) ;
**Levels of Harm according to the NCC MERP index (National Coordinating Council for Medication Error Reporting and Prevention)(9) ; AE: Adverse Event ; LL: Lower Limb; UL: Upper Limb; VTE:Venous Thrombo-embolism
TT^{CR} : Repeated requests of Chest radiographs at least 3 times within 24 hrs; TT^{LL} : request of a Doppler ultrasound of the lower extremities; TT^{UL} : Request of a Doppler ultrasound of the upper extremities

Supplementary file 2: Summary of previous studies addressing the incidence of venous thromboembolism in hospitalized patients

**Authors**	**Khanna et al.**[10]	**Jenkins et al.**[7]	**Assareh et al**[8]	**Khan et al**[9]
**Year of publication**	2014	2016	2016	2017
**Country**	USA	USA	Australia	Ireland
**Type of Study**	Retrospective	Retrospective after intervention	Retrospective	Prospective
**Inclusion Criteria**	Age > 18 yrs<br>Non-surgical<br>Hospitalisation> 48h	Age >18 yrs<br>Non-psychiatric, Non obstetric<br>Hospitalisation > 48h	Age > 18 yrs<br>Non psychiatric<br>Hospitalisation > 48h	Age > 18 yrs
**Number of Admissions**	2,525,068	73,941	3,331,677	12,024
**Thrombo-embolism events(deep venous thrombosis and pulmonary**	n = 6,297	n=667	n = 38,161	n = 48

**embolism)**				
**Age (yrs)**	62.5 ± 20.00	55.7	80.72 % aged ≥ 55 years	60% aged ≥ 60 years
**Sex Ratio**	F : 49.3%	NA	F : 51.9%	F : 66%
**Length of stay (days)**	18.7 ± 19.5	7.4	12 (quartiles: 7–22)	81% ranged between 2 and 28 days
**Medical vs. Surgical**	NA	178/489	6.72 % vs 93.28 %	66% vs 31%(surgical or obstetrics)
**Incidence of venous thromboembolism**	0.25 %	0.90%	1.1%	0.4%
